# A new species of *Ogdoconta* Butler (Lepidoptera, Noctuidae, Condicinae, Condicini) from southeastern Arizona, USA

**DOI:** 10.3897/zookeys.527.9771

**Published:** 2015-10-15

**Authors:** Lars G. Crabo

**Affiliations:** 1 Washington State University Adjunct Faculty; 724 14th Street, Bellingham, Washington 98225

**Keywords:** DNA barcode, Sonora

## Abstract

A new species of *Ogdoconta* Butler (Lepidoptera, Noctuidae, Condicinae, Condicini) is described from the Patagonia Mountains, Santa Cruz County, Arizona, USA. *Ogdoconta margareta* **sp. n.**, is related closely to *Ogdoconta tacna* (Barnes) from Texas. Modifications are proposed to a recently published key to the *Ogdoconta* species north of Mexico to allow identification of the new species.

## Introduction

The North American noctuid moth genus *Ogdoconta* Butler was revised recently by Metzler et al. (2013). There are approximately 16 species in the genus, of which nine occur north of Mexico. The most characteristic feature of the genus is a horizontal cleft in the valve of the male genitalia that divides it into dorsal and ventral components.

A single specimen of a new *Ogdoconta* species resembling *Ogdoconta tacna* (Barnes), a species that is known only from Texas in the United States, was collected in 2013 near Harshaw in the Patagonia Mountains, Santa Cruz County, Arizona. While the new species and *Ogdoconta tacna* are very similar superficially, their male genitalia are distinct and their CO1 barcode sequences differ by nearly 4%. A second specimen of the new species from Sonora, Mexico, was identified by its CO1 barcode and is included in the type series. This new species is described herein.

## Materials and methods

Wing pattern and genitalia structure terminology follow Lafontaine (2004).

The male genitalia were prepared using standard methods (Hardwick 1950, Lafontaine 2004). The detached abdomen was boiled in 10% KOH for 40 minutes. Dissection was performed initially in water followed by hardening with isopropyl alcohol. The male vesica was everted and inflated. The preparation was stained with orcein and was mounted in Euparal on a glass slide.

The 658 base pair DNA “barcode” region of the mitochondrial cytochrome *c* oxidase subunit 1 (CO1) was used to assess molecular variation in the genus *Odgoconta*. A leg of the Arizona specimen was submitted to the Barcodes of Life Campaign (BOLD) at the University of Guelph (Ontario, Canada) where it was analyzed by standard DNA extraction, amplification, and sequencing protocols as described by Hebert et al. (2003). The barcode sequence was compared to pre-existing *Ogdoconta* material at BOLD using the Kimura-2-Parameter distance model as implemented on the Barcode of Life Data Systems website (http://www.barcodinglife.org).

The following collection abbreviations are used:

CNC Canadian National Collection of Insects, Arachnids, and Nematodes, Ottawa, Ontario, Canada

LGC Lars Crabo personal collection, Bellingham, Washington, USA

## Results

### Ogdoconta margareta

Taxon classificationAnimaliaLepidopteraNoctuidae

Crabo sp. n.

http://zoobank.org/3E1BF3CD-CEB1-430E-8F5E-64CDF35248EC

Figs 1 , 2

#### Type locality.

Harshaw, 2.9–5 km SW, Santa Cruz County, Arizona, USA.

#### Type material.

**Holotype**: Male: USA: AZ: Santa Cruz Co., Harshaw, 2.9–5 km SW, 31.42–[31].45° -110.72–[110].74°, 1490–1765 m, 1 IX 2013, L Crabo leg./Crabo [genitalia] slide 681/BOLD CNCLEP 00113651. CNC. **Paratype**: One male: Mexico, Sonora/BOLD CNCLEP 83594. LGC.

#### Etymology.

I take pleasure in naming this species after my mother, Margareta Crabo of Cave Creek, Arizona. The holotype was collected three days prior to her 80^th^ birthday. The name is a noun in apposition.

#### Diagnosis.

**Figure 1–4.**
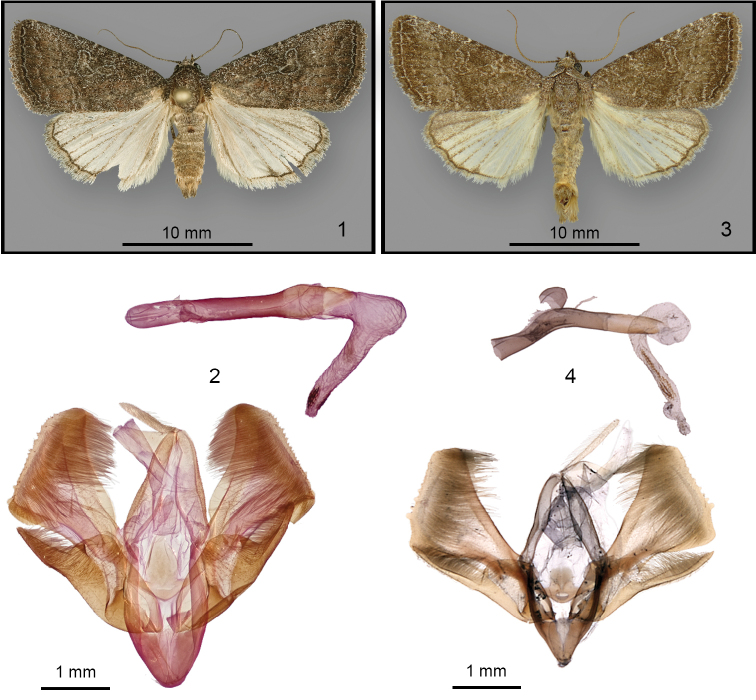
**1** *Ogdoconta margareta*, holotype male (abdomen removed subsequently for dissection) **2** *Ogdoconta margareta*, male genitalia, valves and aedeagus **3** *Ogdoconta tacna*, adult male, Texas, USA (CNC) **4** *Ogdoconta tacna*, male genitalia, valves and aedeagus (source image same as in Metzler et al. (2013), used with permission).

The male of *Ogdoconta margareta* (Fig. 1) resembles the male of *Ogdoconta tacna* (Fig. 3), although the ranges of these species are not known to overlap. *Ogdoconta margareta* occurs in southeastern Arizona, USA and northeastern Sonora, Mexico and *Ogdoconta tacna* occurs in central and southeastern Texas, USA (Metzler et al. 2013).

The valves of both species are unique in the genus in having a broadly triangular cucullus with an irregular outer margin with a series of small knobs. The most prominent structural differences between these species are in the aedeagus, vesica, and sacculus of the valve. In the genitalia of *Ogdoconta margareta* (Fig. 2) the aedeagus is straight and the vesica is straight distal to a basal 135° bend, whereas in the genitalia of *Ogdoconta tacna* (Fig. 4) the aedeagus has a proximal bend and the proximal and distal portions of the vesica are coiled. The dorsal sacculus of *Ogdoconta margareta* is triangular with greatest width near the distal end, whereas that of *Ogdoconta tacna* is rounded with greatest width at the mid-point. The saccular extension of *Ogdoconta margareta* is relatively short, not reaching the ventral cucullus as in *Ogdoconta tacna*.

The female of *Ogdoconta margareta* is unknown.

Superficially, *Ogdoconta margareta* and *Ogdoconta tacna* are very similar and key out together in couplet 6 of the key to species in Metzler et al. (*op cit*.). The forewings of *Ogdoconta margareta* have a violet tint whereas those of *Ogdoconta tacna* are greenish. The hindwing of *Ogdoconta margareta* is paler than that of *Ogdoconta tacna* without significant dark suffusion at the anterior margin. *Ogdoconta margareta* can be distinguished from all of the other *Ogdoconta* species that are known to occur in Arizona, *Ogdoconta cinereola* (Guenée), *Ogdoconta moreno* Barnes, and *Ogdoconta rufipenna* Metzler, Knudson, & Poole, by its uniform purplish brown forewing and pale whitish hindwing. The forewings of the other species are either dark red brown or have lighter areas on the distal wing, and their hindwings are darker. Modifications to the *Ogdoconta* key to species in Metzler (*op cit.*) are given in the Discussion, below.

#### Description.

**Adult male** (Fig. 1). *Head*: Antenna filiform with sparse ventral cilia, dorsum with alternating off-white and gray scales; scape, head, and labial palpus covered with gray, off-white-tipped gray, and sparse off-white scales; frons smooth, unmodified; labial palpus with third segment one-third the length of the second segment; eye smooth, normal sized. *Thorax*: Entire thorax, including prothoracic collar and patagium, covered with off-white-tipped gray and gray scales, appearing uniform purplish brown similar to the forewings; legs with off-white and gray scales, prothoracic leg palest, tarsal segments gray with distal off-white bands. Forewing: length 13 mm excluding fringe; covered with brown-gray, off-white, and fawn scales, appearing hoary purplish brown, slightly darker gray medial to the subterminal line and terminal area and slightly paler near the postmedial line; basal and antemedial lines nearly obsolete, evident as a few pale scales on the costa and in the fold; medial shade dark gray, faint and diffuse; postmedial line brown gray, double with filling of the adjacent ground color, outer portion weakly dentate with dark and pale scales on the veins lateral to the line, oriented parallel to outer margin, nearly straight; subterminal line a hoary sinuous row of pale scales; terminal line dark brown bordered mesially by an incomplete line of pale scales; fringe gray brown with hoary pale tips; orbicular and reniform spots hoary, filled with the adjacent ground color, orbicular spot irregularly ovate, lateral portion touching posterior reniform spot; reniform spot asymmetrically figure-eight shaped with posterior margin extended toward base and touching orbicular spot; claviform spot absent; hindwing slightly brownish off white with slight dusting of pale gray scales near anterior margin and darker gray veins; terminal line dark brown; hindwing fringe pale tan with scattered gray scales and paler outer margin. *Abdomen* (removed for dissection after photography): covered with fuscous scales, slightly darker weak dorsal tufts on the first two segments. *Male genitalia* (Fig. 2): Uncus cylindrical, 6× as long as thick, apex pointed bluntly; juxta 1.25× as tall as wide with arrowhead-shaped caudal portion; valve bifid with larger dorsal portion bearing triangular cucullus and small ventral portion comprised of saccular extension; sacculus 0.5× as long as valve, dorsal margin bluntly triangular and extending to near dorsal valve margin distal to mid-point of sacculus near mid-valve, blade-like saccular extensions 0.4× as long as sacculus, extending to near ventral cucullus margin, asymmetrical, slightly shorter and more robust on the right than the left; dorsal margin of mid-valve slightly convex, cucullus large, triangular, 0.5× as wide as valve length, with finely crenulate lateral margin and rounded dorsal and truncate ventral margins, mesial surface covered with fine hairs; aedeagus cylindrical, straight, 10× as long as wide, without ornamentation; vesica 1× as long as aedeagus with slight expansion at subbasal bend but otherwise similar in width to aedeagus, straight beyond 135° subbasal bend toward left, subapex with two short fields of innumerable short cornuti. **Female**: unknown.

#### Distribution and biology.

*Ogdoconta margareta* is a rarely collected species that is known only from the type locality in southeastern Arizona, USA and Sonora, Mexico.

The two known specimens were collected in early September. It is possible that *Ogdoconta margareta* also flies during the spring because the closely related species *Ogdoconta tacna* has two broods (Metzler et al. 2013).

The holotype was collected in one of a series of black light traps placed mostly in a forest of oak and pine, with a few traps in the ecotone between forest and shrub desert. The habitat of this species in Mexico is unknown.

The early stages are unknown.

#### Discussion.

The Key to the species of *Ogdoconta* in North America north of Mexico in Metzler et al. (*op cit.*) can be modified to include *Ogdoconta margareta* by substituting the following couplet 6 for the original and inserting the following couplet 8 after couplet 7:

**Table:** 

6	Forewing with subterminal area lighter than medial and terminal area; postmedial area usually shaded with pink (specimens from southeastern Arizona with less pink); hindwing of both sexes solidly dark brown	***cinereola***
–	Forewing with subterminal area barely lighter than medial and terminal area; hind wing of male white; hind wing of female pale at base, darker towards outer margin (female of *Ogdoconta margareta* unknown but likely similar)	**8**
8	Sacculus of male valve with rounded dorsal margin, broadest at mid-sacculus; distributed in Texas	***tacna***
–	Sacculus of male valve with triangular dorsal margin, broadest near distal sacculus; distributed in southeastern Arizona	***margareta***

## Supplementary Material

XML Treatment for Ogdoconta margareta
